# Foreign

**Published:** 1841-02-27

**Authors:** 


### FOREIGN.

*Irritation of a Branch of the Trigeminus Nerve at its central extremity.* By DR. BUDGE, of Altenkirchen.—A peasant woman, forty-six years old, was affected for the first time in her sixth year with a severe periodical pain over the right eye, for which she could ascribe no other cause than exposure to cold and damp one winter's day. Previously she had always been healthy, and her parents attained a very advanced age. The pain continued from her sixth to her twelfth year, recurring about every fortnight, lasting from twenty-four to thirty hours each time, and then leaving her again well and hearty. When she was twelve years old it ceased; and during the next twelve years never once returned. In the interval she commenced and regularly continued menstruating. But at the end of this time, when twenty-four years old, she had a child, and eight days after delivery her old pain returned; from that time, now twenty-two years ago, she has never been a week without it.

The pain usually begins at midnight, awakening the patient from sleep, and lasts to the same time in the second night following, i. e., for about forty-eight hours. It is preceded and accompanied by chilliness, which at last terminating in sweat, indicates the approach of the end of the attack. The pain extends from the lamdoidal suture of the right side, and is most severe at the posterior and anterior surfaces of the upper eyelid, about half an inch above the brow, and a short distance below the eye of the same side. It is a stabbing pain, and so intense that the mere thought of it fills the patient with horror; it continues uninterruptedly through its whole period, and shortly before it ceases attains an indescribable severity. The slightest touch excites intense agony in the affected part. The immediate neighbourhood and all the rest of the face are as to sensation perfectly natural; but the features on the right side are less marked than on the left side of the face. The cornea (during the pain) is rather turbid, and the eye is filled with tears, the sclerotica is reddened, and the vessels of the conjunctiva turgid; the tarsi are also swollen and red. The whole eye is turned outwards, so that the cornea is nearly hidden; and the patient can with difficulty look straightforward with it, partly from loss of power over it, and partly because the straight position, if retained but for a few instants, excites giddiness and vomiting.

Soon after the commencement of the pain, giddiness comes on, and compels the patient to keep her bed; it increases with the pain, and usually lasts a day longer than it does. The patient is at the same time constantly tormented by sickness and vomiting of food, mucus, and bile. Her tongue is quite clean and her bowels regular, but she has no appetite. The organs of the abdomen and chest and the pulse are in a normal condition.

The first and second cervical vertebræ are a little sensitive on pressure, and from the third to the second dorsal there is extreme sensibility, especially over the third, fourth, fifth, and sixth cervical. As often as these are struck the patient flinches and complains of pain in the right eye, and immediately after vomits. The right arm also, which sometimes moves spontaneously when the pain is very intense, is sometimes convulsed when these vertebræ are pressed.

When the paroxysm is about to cease, the intensity of the pain, the squinting, and the giddiness decrease, and soon after a general sweat comes on. At the last the patient sneezes once or twice (and always, she says, through the right nostril) and then, except for the giddiness, which lasts a day longer, the disease is for the time removed. Every fresh attack, however, is severer than the preceding, in respect both of the pain and of the squinting. The paroxysms are rarer in the summer than in the winter and spring; mental anxiety and passion accelerate and aggravate them, but neither menstruation nor pregnancy has any influence on them.

In the intervals between the attacks of pain, there is no external appearance of illness whatever, except a slight outward squint; and, but for the influence of pressure on the spine, the woman might be deemed perfectly healthy. By this, however, though there is never any spontaneous pain in the back, all the signs of the disease may be in a slight degree voluntarily reproduced; as soon as the lower cervical vertebræ are pressed, the patient complains of the pain of her eye and head, the right eye turns outwards, tears flow into it, and sickness or even vomiting comes on. And again, all these phenomena cease as soon as the pressure is remitted.

Of the above singular symptoms, the author offers the following explanations:

The first and most important, the pain over the right eye, is evidently the result of a state of irritation of the majority of the fibres of the frontal branch of the trigeminus, whose exact course it follows; for the patient says that the pain is not in the eye itself, but passes above it from behind forwards, and extends upwards and a little inwards towards the nose. The pain in the back further shows that the exact seat of this irritation is the central extremity of the nerve, of which, as is well known, the roots have been traced to the region of the third and fourth cervical vertebræ, and probably go down still lower. Immediately over this part, the patient has pain on pressure: and by it the pain in the eye may be produced and increased at will, although, except during the paroxysm, no more pain is excited by pressure on the right than by that on the left eye.

The pain in the other adjacent vertebræ probably results both from the stimulus of pressure on the extremities of the nerves over them, which from their proximity to the nervous centre are especially liable to exalted excitability, and from the immediate effects of the pressure on the membranes which are themselves excited and sensitive in an unnatural degree.

The drawing of the eye outwards is the result of an influence on the sixth nerve; an influence reflected from the sensitive nerves. It is not probable that the central ends of the fibres of the sixth are directly irritated; for if that were the case, the same extremities of the other nervous fibres that lie between the sixth and the fifth would most likely be also irritated, and the morbid phenomena would be more extensive. The twitchings of the right arm may also in like manner be regarded as reflex motions.

The organic alterations in the eye must be considered as signs of the excitement of the trigeminus; and as when a sensitive nerve is divided, nutrition is materially impaired, and the part it supplies undergoes a kind of maceration, so when one is irritated the same part is inflamed.

The giddiness is thus explicable; fibres of the trigeminus may be traced into the crura cerebelli ad frontem, near the spot from which the nerve comes out, and as Magendie has shown that when these crura are divided, the balance of the body seems to be lost, it is clear how an irritation of the trigeminus induces giddiness, and how that symptom is most marked when the signs of irritation are passing off or gone.

The vomiting is explained partly by the affection of the spinal cord and partly by the influence of the nerves on the sight. (In explanaton of this the author refers to his own investigations on vomitings recently published.)

The shivering is the result of the stimulus of the central extremities of the sensitive nerves in the spinal cord; and it is comparable with the results of several of the author's experiments, in which he has observed that on exposing the spinal cord in a dog or other animal, and gently stroking it with a feather, all the characters of a shivering fit are immediately produced. In this, as in all other cases, the condition of the central extremity of the nervous fibre is felt as if it were that of its peripheral extremity.

The sneezing at the end of the paroxysms probably results from the nasal branch of the trigeminus becoming involved in the excitement when the irritation of the frontal is at its greatest height. The excitement is in this case also probably communicated in the nervous centre, though felt where the fibres are distributed; and it is communicated not to the sensitive nerves alone, but by reflex action to the nerves of the respiratory muscles.

The author, believing that the essential cause of the symptoms was a congestion of the vessels of the frontal nerve, had ten cupping-glasses applied every eight days near the vertebræ at which pain was produced by pressure; these were applied three times; mercurial ointment was rubbed in morning and evening, and four grains of sulphate of quinine with two of extr. nucis vomicæ were given in two doses every day. Their result was most striking; the patient, who had for twenty-two years been entirely unrelieved by a variety of methods of treatment, had no severe attack after this was employed; after several months had elapsed, she continued well, and even the divergence of the right eye was in some degree lessened.—*Brit. and For. Med. Rev.*, *from Casper's Wochenschrift. Oct.* 3, 1840.

---

*Case of Idiopathic Lymphadenitis.* By Dr. Pogliaghi, of Milan.—C. C. aged forty-eight, a man of robust constitution, but of very debauched habits, after a few days' illness, was attacked by a troublesome inflammation, which appeared first at the sides of the face, extending from the front of the ears along the margin of the lower jaw, and meeting together at the chin. Emollients were used without benefit, and fever coming on, a physician purged and bled him twice. Being in some measure relieved by these means, the patient neglected himself and suffered the disease to make slow though scarcely perceptible progress. After some days of apparent calm, small almost painless tumours appeared at the lower part of the neck; to these succeeded others in the axillæ and the groins, and, after these, fresh ones in the hams and at the bends of the elbows; all of which in the course of three days had increased so as to render the movements of the joints inconvenient. The patient now resorted to various quack medicines, and at last growing worse was sent to the infirmary, having been ill altogether about seven weeks.

At first sight it was supposed that he had the disease known by the name of *orecchioni* or of parotiditis rheumatica, for his face presented at either side a slightly painful swelling, commencing in front of the meatus auditorius externus, following the lower margin of the jaw, and meeting that from the opposite side at the chin; and feeling as if it were formed by an agglomeration of divers lobes. The further examination found some tumours as large as pigeons' eggs at the base of the neck, others much larger in the axillæ, others as large as those in the neck at the elbows, and again others as large as a strong man's fist in the groins, and of various sizes in the hams. The tumours on the left side were generally larger than those on the right; the skin covering them all had its natural colour, and there was no increase of heat. Motion of the joints was painful. The patient had slight fever and rather a full pulse; his face was livid red, and his eyes prominent as if he had a ligature on his neck; his head was heavy, his tongue coated, his appetite not destroyed, his respiration free but accompanied by frequent sighing, his abdomen presenting nothing morbid.

The patient was bled nine times, (for reasons which are given in detail,) leeches were twice applied to the neck, and mild purgatives, followed by pills containing calomel and extract of aconite, were given. After the first bleeding, (from which as from all the rest the blood was buffed,) the patient seemed somewhat relieved, and the tumours appeared less hard; but subsequently the fever became more severe, and had regular evening exacerbations, and the patient had frequent attacks of dyspnœa. At first these were slight and occurred most frequently in the night, but afterwards they became more severe and prolonged, and in one of them he died suddenly, nine days after his admission into the hospital, and about two months from the commencement of his illness.

At the examination of the body, some of the enlarged glands were found in a state of incipient inflammation, others with points of beginning suppuration at their centres, and others transformed into cysts containing a substance of the consistence of cream and of the colour of dregs of wine. In some of these last the cavity was as large as a pigeon's egg, and in two in the axilla it would have contained a hen's egg. The alteration had advanced furthest in the glands of the left axilla, less in those of the left groin, and still less in those of the right groin. The thoracic viscera were healthy except for a slight serous effusion in the right pleura; but the glands at the bifurcation of the trachea were enlarged to the size of a big fist. Internally they presented the various degrees of the inflammatory process observed in the other glands, though in a less advanced state: some presented points of suppuration, but the matter had not the lurid wine-dregs-colour seen in the glands of the external parts of the body. In the abdomen nothing anormal was found except incipient inflammation of the mesenteric glands.—*Ibid., from Annali Universali di Medicina. Marzo*, 1840.

---

*On the Utility of Graduated Compression of the Abdomen by Bandages, in the Treatment of Ascites.* By Professor L. Morelli, of Siena.—From among many cases that were either cured or materially relieved by this mode of treatment, the author relates one in which its benefit was most strikingly shown. It was that of a woman upwards of fifty years old, who from poverty, and other sources of misery, had become gradually more and more ill, and had at length come to the Clinical Hospital with ascites from diseased liver. She had been salivated, and had undergone a variety of treatment, and had been tapped four times. The author recommended his system of bandaging, but for a long time the patient refused to adopt it, and in the interval it was found necessary to perform paracentesis eight times more. After the twelfth operation the patient, now reduced to a most perilous state, agreed to submit to the bandaging, of which she had seen the good effects in a dropsical child. The best kind of bandage consists of a band extending from the lower third of the sternum to the pubes, and round to the loins, and there gradually tightened by means of strips of leather, with several holes in them for the passage of the points of small pins. The whole is prevented from shifting upwards by two strips of strong cloth, which pass downwards, and are stitched behind in the neighbourhood of the sacro-iliac symphyses. This apparatus being put on, and gradually tightened, the abdomen of the patient slowly returned to its natural size; the urine flowed abundantly, her appetite and sleep returned, and, at length, after nearly three years' illness, in which she had used a variety of medicines, and had been tapped twelve times, she was restored to good health. A year afterwards, having discontinued the bandage, the ascites appeared again in a slight degree, but purgative medicines and the reapplication of the compression again speedily removed it.

At its first application, and for some days afterwards, the bandage produces great inconvenience; so much, that many patients refuse altogether to wear it for a sufficient length of time; but by a proper selection of cases, and careful management of them in other respects, the author asserts that compression will be found one of the most successful means that have ever been employed against ascites. It must be confessed, however, that he gives no indications, nor does he himself seem to have learned in which of the very different circumstances in which ascites occurs, his method is either applicable or likely to prove useful.—*Ibid., from Annali Universali di Medicina. Marzo*, 1840.

---

*On a Sedative Lotion in Headachs, Congestions, and Cerebral Fevers.* By M. Raspail.—Professor Raspail, in a letter to the editor of *l'Expérience*, gives the mode of preparing a lotion, the sedative effect of which, he says, is almost instantaneous. It is as follows:

| | |
|---|---|
| Liquor of ammonia (Qy. the strength?) | 100 pts. |
| Distilled water - - - - | 900 " |
| Purified marine salt - - - - | 20 " |
| Camphor - - - - - | 2 " |

Essence of rose, or some other scent, in the necessary proportion.
The whole dissolved cold.

A piece of linen is to be steeped in this solution and applied over the part of the head that the patient points out as the seat of pain, taking care, if it is on the forehead, to apply a thick bandage over the eyebrows, to prevent any drops of the fluid passing into the eyes.

M. Raspail says he has seen headachs intolerably violent, accompanied by photophobia, and retraction of the globes of the eye, disappear completely, from a quarter to half an hour after the application of one wetted cloth. The linen is to be soaked as often as a new access of pain is threatened, and left on the head until it is necessary to soak it anew. In the numerous trials the author has made with this solution, first on himself, and afterwards on others, he has been struck by two circumstances of interest in connexion with organic chemistry and symptomatology. When in a violent attack of cerebral fever, we apply on the principal seat of the inflammation a concentrated solution of marine salt, an evident odour of chlorine is disengaged, the diseased reaction being analogous to the decomposing and deoxygenating action of manganese, in the elimination of chlorine from marine salt, by means of sulphuric acid. Is this sign constant in affections of this class? It is for experience to decide. When, on the contrary, we employ a solution of ammonia, a strongly characterized *hircine* (goatish) odour is manifested. The same odour has been disengaged on the application of hydrochloric acid to the skin. M. Raspail has drawn the attention of the profession to this subject in order that they may employ this formula, and fix their attention on the analysis of the disengaged substances, as they may become characteristic of special affections. —*Ibid., from l'Expérience.* 24 *Juillet*, 1840.

*Case of Posthumous Variola.* By Dr. Chansarel.—A girl, aged five years and eight months, who had been vaccinated, was suddenly attacked, when in perfect health, with a varioloid eruption and fever. About twelve hours afterwards, the redness of the skin and the pimples totally disapeared, cerebral phenomena supervened, and death twenty-six hours after the first appearance of illness. On the next day, thirteen hours after death, "I saw," says M. Chansarel, "to my great astonishment, a great number of pimples which had appeared after death, and which were much more developed than those I had seen during the life of the child. They were seated on the face, neck, chest, and buttocks; those in the latter region being larger, equalling the size of a lentil. They had the aspect of variolous pustules, surrounded by a red areola, depressed in their centre, and contained a small quantity of fluid. The body exhaled a fetid odour, and exhibited some red and livid spots, putrefaction not having been so slow as it appeared."—*Ibid., from Bulletin Medical du Midi. Juin, Juillet*, 1840.

*Mode of Resolving Engorgements of the Spleen.* By M. Voisin, of Limoges.—The author has employed the following treatment with success in eight or ten cases of these engorgements after intermittent fevers. In three or four of these cases the diseased organ occupied about two-thirds of the left half of the abdomen. The treatment is simply to apply a mercurial plaster (*vigo cum mercurio*) with which is incorporated six or eight scruples of the sulphate of quinine, more or less. It is to be renewed when the matter of which it is composed is exhausted, that is, from forty to fifty days. The advantages of this mode of treatmen. are: 1. It saves the patient from the disgust which he undergoes when the quinine is administered by the mouth. 2. The absorption and consequent action of the remedy are continued. 3. This absorption and this action are accomplished in the immediate neighbourhood of the diseased organ. 4. Owing to the continuance, the fever does not reappear.

This treatment has alone sufficed for the cnre. The period of cure will vary as the spleen is more or less engorged, the patient more or less aged, and the skin more or less readily absorbing. Ordinarily, two or three months suffice.—*Ibid., from Gazette Medicale de Paris.* 12 *Septembre*, 1840.

*Development of Acute Glanders by the Introduction of Pus into the Veins of a Horse.* By M. Renault.—M. Renault presented to the Academy of Medicine some anatomical specimens taken from a horse in which acute glanders was developed by the introduction of pus into its veins. The pus was taken from an abscess on the breast of a horse four years old, perfectly free from any glanders or farcy. The animal experimented upon was a small horse of a rustic breed, lean, but in good health, high spirited, and of good appetite. An emulsion was made with four centilitres of pus in ten decilitres of distilled water. This emulsion filtered through fine linen was introduced into the veins of the horse on the 13th of July. The next day a very intense fever manifested itself; the heart beat ninety in the minute; three days afterwards a pustule appeared in the left nostril, and a pustular eruption showed itself on the chest; on the following days the acute glanders was shown with all its characters. The animal died on the 20th of July.

On a post-mortem examination there appeared ulcerated pustules on the surface of the pituitary membrane, and purulent infiltrations in its substance; in the lungs there were many metastatic abscesses and lobular pneumonia; and in the spleen and walls of the heart purulent granulations were found.—*Ibid., from Bulletin de l'Académie Royale de Médecine.* 15 *et* 31 *Août*, 1840.

*Subcutaneous Section of Forty-two Muscles, Tendons, or Ligaments, practised the same day, on the same person, to remedy a general articular deformity.* By M. Jules Guerin.—In a letter addressed to the Academy of Sciences on the 31st of August, 1840, M. Guerin states that on the 25th of that month, he performed the section of forty-two tendons, muscles, or ligaments, to remedy a series of articular deformities of the trunk and limbs, caused by the active retraction of these muscles and ligaments. There were twenty-eight openings of the skin required. The followings were the parts divided:

| | | | |
|---|---|---|---|
| Trunk | Pectoralis major | | 1 |
| Arms | On each side | biceps cubiti | 2 |
| | " | pronator teres | 2 |
| | " | extens. carpi radialis | 2 |
| | " | flexor com. sublimis | 2 |
| | " | palmaris brevis | 2 |

| | | |
|---|---|---|
| Forearms | Tendons of extensor carpi ulnaris on each side | 2 |
| | " palm. longus & brevis | 4 |
| | " abductor pollicis | 2 |
| Legs | The sartorius on each side | 2 |
| | The biceps cruris | 2 |
| | The semi-membranosus | 2 |
| | The semi-tendinosus | 2 |
| | The rectus femoris | 2 |
| | Fascia lata | 1 |
| | External lat. ligaments of knee | 2 |
| Feet | The tendo Achillis on each side | 2 |
| | The tibialis anticus | 2 |
| | The extensor communis | 2 |
| | The extensor proprius pollicis | 2 |
| | The peronei antici | 2 |
| | | 42 |

The patient only experienced moderate pain or fatigue, and did not complain during the operations, which lasted an hour. An hour afterwards he was in a sound sleep. He was very tranquil the following night and day. No inflammatory accident supervened, and, on the third day, the twenty-eight wounds were completely cicatrized. The sections were made in the presence of many distinguished French and foreign surgeons. M. Guerin holds that he is not open to the charge of rashness, as he first established the absolute innocuousness of subcutaneous wounds by numerous experiments on animals, and then verified the same principle in man by a series of operations from the section of one muscle to that of a great number. He purposes to lay an account of his method of operating with its definite results before the Academy in a future number.—*Ibid., from Gazette Médicale de Paris. Septembre* 5, 1840.

*On a New Species of Torticollis.* By M. Bouvier.—This species of torticollis differs altogether from the acute or chronic muscular form, and from the spontaneous luxation of the atlas and axis, three states with which it is been confounded. It has its seat in the articulations of the first cervical vertebræ, and may perhaps be designated by the term articular torticollis. The lateral articulations, right or left, of the first cervical vertebræ, and especially of the atlas with the axis, are, so to speak, the focus of articular torticollis, which consists in a peculiar form of inflammation of the synovial capsule and fibrous tissues of these articulations. The essential characters are: 1. A position of the head and neck, similar to that produced by contraction of one of the sterno-cleido-mastoid muscles, namely, lateral flexion to the right or left, and rotation of the face to the opposite side. 2. A pain felt towards the top and sides of the nape of the neck, sometimes on the lateral and posterior parts of the cranium; arising spontaneously, or in the movements of the neck. 3. Complete relaxation of the sterno-cleido-mastoid of the affected side, and an equal tension of the muscles of the neck both left and right. 4. Stiffness and pain in all or any of its movements, notwithstanding the preservation of the articular connexions of the cervical vertebræ, and the integrity of the muscles. 5. Absence of swelling, and of sensibility on pressure. 6. Atrophy, or arrest of development of the side of the face, which answers to the inclination of the head, when this has lasted a certain time.

This disease prevents two successive states or periods, the acute and chronic. It is often produced by cold, sometimes by sudden distension of the ligaments. M. Bouvier thinks it very important to detect this disease, as the section of the muscle would be useless and rash, but reserves to another occasion the proceedings which he has found useful.—*Ibid., from l'Expérience.* 17 *Septembre*, 1840.

*Wound of the Urinary Bladder.*—By Dr. Schûtte, of Mullheim.—A healthy man, thirty-seven years old, fell perpendicularly from a height of about eight feet, on an upright wooden stake several feet long and fully an inch thick. Its end passed into the inner surface of the left thigh, about three inches from the rectum, and ran into the lower part of the urinary bladder above the sphincter vesicæ. The urine flowed continually and insensibly through the wound; but neither blood nor urine passed through the urethra. A catheter was pleced in the urethra, and several leeches and lotions of cold water were applied externally; and when the danger of severe inflammation had passed by, bread and water poultices were put over the wound. The patient was allowed only mild food, and in about three weeks the wound had healed without any ill consequences.—*Ibid., from Medicinische Zeitung. October* 7, 1840.

*On a new method of Operating for the radical cure of Hernia.* By M. Velpeau.—The author of this memoir, after examining at length the various means hitherto employed for the cure of hernia, states that he finds none of them at all efficacious; he therefore determined to try another method. Convinced from repeated experiments that injections with tincture of iodine produced in the serous membranes an adhesive inflammation, slight and scarcely dangerous, he attempted the radical cure of hernia by these injections. Three patients were operated on by him in the years 1836 and 1837, but the difficulties of a first operation, and the vagueness of the results, still left him in doubt, and he afterwards employed a method in two cases consisting of three distinct steps: subcutaneous puncture, scarification of the interior of the sac, and compression. He took the idea of scarifications from the ancients, or rather from what he had himself advanced in the year 1832. Twice that year he had placed great confidence in them, and only discontinued their employment when he perceived that danger was likely to ensue to the patient from opening the sac. These dangers seemed no longer likely, when M. Guerin gave the hint of the possibility of penetrating to the bottom of the cavity, by a simple puncture of the integuments, and expressed in his memoir his desire of applying his subcutaneous incisions to the radical cure of hernia. M. Velpeau has performed the necessary scarifications in this manner. With regard to the compression, he applies it not only to the external abdominal ring, but over the internal ring, and in the inguinal canal itself. He employs the celebrated bandages of M. Fournier of Lempdes, which he states have more than once sufficed for the radical cure of hernia.

M. Velpeau successfully operated on a patient in the following manner: Having introduced the index-finger for some lines in depth into the external abdominal ring, he pushed the integuments before it, and passed a sort of lancet over the nail of this finger, pushing it obliquely as far as the iliac fossa; then withdrawing his finger from the ring, he brought the edge of the instrument against the iliac walls of the abdomen, which he supported with the other hand, and then scarified them in various directions, taking especial care to avoid the epigastric vessels. The lancet was then withdrawn at the exact spot where it had entered, and the operation was complete, only a few drops of blood having been lost. The patient was taken to bed, no accident occurred, the small wound cicatrized by the next day, the bandage of Fournier was applied on the third day, and the man has since walked and conducted himself as if he had never been afflicted.

Encouraged by this result, the patient demanded a similar operation on another hernia. It was performed, and at the end of ten days he was convalescent. He has since continued quite well, and has remained as a cook in the hospital to show the radical cure to the profession.—*Ibid.*, *from Bulletin Géneral de Thérapeutique.* 15 *et* 30 *Août*, 1840.

---

*On Hydrophobia and the relation between the Retina and the Respiratory Nerves in that Disease.* By Dr. A. Triberti, Physician in Ordinary to the *Ospedale Maggiore* of Milan.—The main object of this paper is to recommend that hydrophobic patients should be kept in almost perfect darkness, and carefully excluded from the view of any thing bright or glistening, to avoid the severe paroxyms which the sight of such objects has never failed to bring on in all the cases that the author has seen. These have been seventeen in upwards of thirty years' practice. In all these cases the hydrophobia did not once come on before the 25th, nor after the 60th day from the reception of the bite. All were hydrophobic when they were brought to the hospital, and in all, the wound which had cicatrized a few days after the bite, was found red, with heat, burning, and a painful and spasmodic sensation of the whole limb, or of all the bitten part. In five of the seventeen cases the wound had opened again, and presented a malignant ulcer, discharging a fetid serous fluid. All died on the second or on the third day from the commencement of the attack. All complained of languor and lassitude, which were accompanied by anxiety and deep sighing; all were desirous of being left alone, and in the dark; in all the pulse was quick and irregular, and in some rather full. After the first twenty-four hours of their being in the hospital, the patients all complained of tightness and oppression of the præcordia, with dyspnœa and excessive thirst, which they could not alleviate by drinking fluids, though they could swallow small pieces of solid food. The spasms of the muscles of the pharynx produced in all the patients a distressing sensation of a ball or globe in it, which was always increased at the sight of any drink, but was diminished by swallowing any solid substance. The spasm of these muscles was also in all cases accompanied by convulsive movements of the head, and by severe trembling of the other muscles of the neck.—*Ibid. from Annali Universali di Medicina.*

---

*On the Employment of large Doses of Tartarised Antimony in the Treatment of Articular Dropsies.* By M. Gimelle.—M. Gimelle who some time ago published a series of facts, illustrative of the utility of tartar emetic in hydarthrosis, has continued his observations, and finds that when an articulation is the seat of synovial effusion, the same treatment is the most effectual for procuring speedy resorption. In twenty-eight cases the emetic tartar was given in increasing doses, commencing with four grains in the twenty-four hours, and increasing two grains daily till the dose was sixteen, eighteen, or twenty grains per diem, with the invariable effect of determining the resorption of the liquid in a space of time, varying from eight to sixteen days. Of twenty-eight cases of effusion of synovia into articulations, twenty-two had their seat in the knee-joint; three were double, and two were in the shoulder-joint; one was in the elbow, and one in the ancle. All the patients took the emetic tartar in infusion of linden tree with syrup of poppies. In eighteen the tolerance was established on the first day, in two on the second, and in two on the third. No accident occurred to any of the patients after the tolerance was established. The dose of twenty grains was never exceeded, and in all the cases the effusion was absorbed in the space of eight, ten, or sixteen days—longest period during which the remedy was administered. In twenty-five cases the pain and stiffness felt in the affected articulations diminished at the same time and degree with the effusion, and when the latter had disappeared, the patients could walk as easily as bsfore they were attacked by the disease. In two cases, however, though the liquid disappeared in the ordinary time, the pain remained in one case for a month and in the other for forty days. In one case the remedy was carried to twelve grains without benefit, and as it was one of very old standing, it was thought proper to relinquish the medicine.

We give the following cases, as they are very interesting proofs of the value of the treatment employed.

CASE I. A man, aged seventy-three, was affected by a very large hydarthrosis of the left knee, which extended into the hollow of the ham, where it formed a tumour of the size of the fist, which disappeared on strong pressure, and became visible on each side of the patella. M. Pasquier prescribed the emetic after the form of M. Gimelle. The dose was successively carried to 16 grains; the patient experienced no inconvenience, his appetite continued good, and on the sixteenth day all the signs of effusion into the articulation had disappeared. Two days afterwards the patient was dismissed entirely cured.

CASE II. On the 10th of September M. Gimelle was called to a student, aged twenty-one years, with an hydarthrosis of the right knee, which had been treated without success during six weeks by leeches, blisters, compression, frictions, and embrocations. M. Gimelle prescribed the tartar emetic. The patient's appetite continued good, and he had no inconvenience. The dose of twelve grains was not exceeded, and fourteen days after this treatment was commenced there was no trace of synovial effusion, the patient feeling merely a feebleness of the limb.

CASE III. A healthy female, aged twenty-three, in a journey from Tours to Paris, caught cold during the night, and, on her arrival in the capital, experienced pains in the right knee. M. Gimelle considered this case one of commencing hydarthrosis, and prescribed without delay a gummy potion with four grains of tartar emetic and an ounce of syrup of poppies. Five or six vomitings, and afterwards alvine dejections followed, but the pain was relieved in the night and the patient could move the limb. On the second day the catamenia appeared, and the emetic was suspended for five days, during which the pain reappeared and the effusion increased, and the articulation became very red and hot. The emetic was then resumed, four grains being given on the first and second day; it produced three or four vomitings, and as many stools. Tolerance was established on the second day, there was a diminution of the synovial tension, and the patient could bear slight movements of the limb without much pain. On the third day six grains were given, eight on the fourth, ten on the fifth, twelve on the sixth, and fourteen on the seventh; the tolerance continued, and the amelioration was progressive. On the eighth, ninth, and tenth days, sixteen grains were given, and on the tenth day the synovial effusion had completely disappeared.

In none of these cases did M. Gimelle precede the employment of the tartar emetic by local or general bloodletting. Nevertheless, he thinks that if the fever be intense, and the articulation present great heat and redness, or if there be great irritation of the digestive organs, it would be proper to combat these symptoms before administering the emetic tartar. By this preliminary treatment we should diminish the chances of gastric irritation, probably facilitate the tolerance, and consequently the action of the remedy.

The most constant effects of the tartar emetic were diminution in the force and rapidity of the pulse, enfeebling of the voice, fatigue and coloration of the eye-lids (known by the name of *yeux cernes,*) and abundant perspirations during the night. Five patients had vomitings, two during one day, one two days, and two during three days. Eight had very abundant alvine dejections, lasting from one to three days: in three the vomiting and purging coexisted. Sixteen experienced neither vomiting nor purging. In the majority the appetite was unaltered; and in those cases where it was disturbed, it was re-established with the tolerance. The quantity of urine was diminished, which M. Gimelle attributed to the abundant perspirations. All the other functions were performed as in the healthy state. The quantity of food the patients had taken when in health was not diminished, and often increased. Lastly, M. Gimelle saw all the patients some months after treatment, and many of them after some years, and in none did any accident occur.—*Ibid., from Bulletin Général de Thérapeutique.*

---

*Experimental Researches relating to the Mode of Transmission of Rabies.* By M. BRESCHET.—At the sitting of the Royal Academy of Sciences, September 21, 1840, M. Breschet brought forward proofs of the contagious nature of hydrophobia. Horror of liquids, and other hydrophobic symptoms, have been observed in man independently of contagion, but they essentially differ from the rabies of inoculation. The term hydrophobia is not applicable to animals, as in them the horror of water is not an essential symptom. Rabies is always produced by inoculation, and MM. Breschet and Magendie proved that it may be transmitted from man to the dog by inoculating a dog with the saliva of a hydrophobic patient. Thirty-eight hours afterwards the dog became furiously rabid; he bit several other dogs, and these became successively rabid. Some of these observations appear to show that the virus becomes enfeebled, or loses its deleterious properties in passing through several persons.

The period of latency of the poison is about twenty or thirty days. Some of the dogs drank water with avidity. The virus is transmitted from carnivorous to herbivorous animals; in an ass bitten by a mad dog the disease was evidenced in its most intense degree. Two horses inoculated by the saliva of a dog had the disease less marked. The saliva of the ass above mentioned was put under the skin of several dogs and rabbits, and they all became rabid. Birds were killed by inoculation, but did not exhibit symptoms of rabies.

Is the blood altered in rabies? M. Breschet answers that he has several times transfused the blood of a rabid into the veins of a healthy dog, but has never determined the development of rabies in this manner. It appears that the saliva of the diseased animals alone affords the necessary conditions for the transmission of rabies. The saliva, or slaver, is really an altered fluid, a humour in a true morbid state; or the vehicle of a deleterious principle, of a true rabic virus newly secreted, but the nature of which is as yet completely unknown to us. Rabies, then, is a virulent contagious disease, and not the effect of a moral affection.—*Ibid.*, *from Archives Génerales de Medecine.*

---

*Solution and Absorption of Provisional Callus during Typhus Fever.* By Dr. Schilling.—An artilleryman received a fracture of the left femur on the 1st of September, and by careful management the ends of the bone were so firmly united in the middle of November, that he could bear some weight on the foot. Symptoms of typhus abdominalis, however, then set in, and ten days afterwards callus could no longer be felt, and the bones moved as freely and as easily upon one another as at the first examination after the reception of the injury. In six days more the patient died. The examination exhibited no trace of callus; the broken surfaces were bloody, like those in a recent fracture, and were surrounded by a saclike membrane, which contained some black bloody fluid.—*Ibid., from Medicinische Zeitung. September* 16, 1840.

---

*On a stony Concretion in a Human Nose.* By Stefano Grandoni.—A woman of the province of Brescia, aged thirty-two, was brought to the hospital for a disease of six months' standing, consisting of a calculus, which was situated in the left nostril. Of this affection she was entirely cured by the extraction of the calculus, which was effected with a pair of polypus-forceps. The concretion, in the condition into which the operation had reduced it, was in fragments of unequal sizes, covered with small rough prominences, furrowed longitudinally, and invested by a kind of crust inseparably united to its chief mass, slightly tinged of an iron-rust colour, and beset with minute pits. The fracture of this substance showed that the texture of the whole mass was very similar to that of the compact calcareous earth, in which there is some crystalline glittering. The concretion had a specific gravity of 1.4, and therefore sank in water; at the ordinary temperature it did not emit any odour; it weighed altogether seventy-six grains. In none of the fragments could there be recognised the nucleus of the concretion, unless one could regard as such a little seed of some grass which occupied the centre of the largest fragment. On being subjected to an accurate chemical analysis, (the details of which are given,) a hundred parts of the calculus yielded the following constituents:

| | |
|---|---|
| Phosphate of lime | 55 |
| Carbonate of lime | 18 |
| Carbonate of magnesia | 7 |
| Organic matter, with traces of iron | 20 |
| | 100 |

*Ibid., from Commentarii dell' Ateneo di Brescia.*

---

*A Case of Spinal Disease, in which hiccough, being one of the most distressing symptoms, was completely removed by inserting a seton over the origin of the phrenic nerves.* By J. Watmough, M. D.—Miss H., last October, whilst dressing a sore on the hand of a poor woman, was suddenly seized with syncope, which caused her to fall on the rugged floor of a cottage; a slight convulsion followed, described by the attendants as lasting for three or four minutes. On coming to herself, she complained of pain in the back, with numbness of the legs.

Two days after, I found her labouring under severe pain in the inferior dorsal and superlumbar vertebræ, with headach and loss of sensation and motion in the lower extremities. Leeches, bleeding from the arm, with small doses of calomel, and counter-irritation on the spine, were the means used. She cannot now, eight days from the first seizure, be moved from the horizontal position; bowels are regulated by purgatives; and slight incontinence of urine relieved by tinct. lyttæ.

Three weeks have now elapsed since she was first attacked. Singultus, which has occurred frequently, now becoming severe, cupping, leeching, blistering, with four caustic issues on the spine, were now had recourse to, together with the internal and external use of preparations of iodine, small doses of strychnia, &c. Notwithstanding, six months passed away without much alleviation of symptoms: singultus became intolerable, threatening to prove fatal rapidly. I inserted a seton over the origin of the phrenic nerves, and eight days after, these formidable symptoms ceased, sense of feeling and power of motion were restored to the legs, and, by the use of the inclined plane, with tonics, &c., my patient gradually progressed to sound health, and is now able to walk well, and travel considerable distances.

*London Medical Gazette.*

